# ENDS consumption in students of higher education: Phenomenon on the rise during the COVID-19 pandemic

**DOI:** 10.18332/tid/154970

**Published:** 2022-11-14

**Authors:** Diana C. Maldonado Viasús, Gustavo A. Perdomo, María C. García Duran, Adriana C. Pulido, Manuela Lüchau Hernandez, Elizabeth Borrero Roldan, M. Catalina Botero, Sergio M. Moreno

**Affiliations:** 1 Epidemiology Department, Population Health Axis, Fundación Santa Fe de Bogotá, Bogotá, Colombia; 2 Mental Health Department, Population Health Axis, Fundación Santa Fe de Bogotá, Bogotá, Colombia; 3 Clinical Mental Health Sciences Department, Population Health Axis, Fundación Santa Fe de Bogotá, Bogotá, Colombia; 4 Public Health Department, Population Health Axis, Fundación Santa Fe de Bogotá, Bogotá, Colombia; 5 Health Psychology Department, Population Health Axis, Fundación Santa Fe de Bogotá, Bogotá, Colombia

**Keywords:** ENDS, e-cigarettes, lifestyle, college students, COVID-19

## Abstract

**INTRODUCTION:**

The COVID-19 pandemic triggered transformations in the population’s lifestyles, including electronic nicotine delivery system (ENDS) consumption. The aim of the study was to determine associations between ENDS consumption habits and lifestyles among higher education students in Bogotá, Colombia.

**METHODS:**

This study employed a cross-sectional analytical design, based on a self-administered online survey, conducted in 2021, among students aged 18–59 years. The sample size was 3985 students. Statistical analysis was done through frequency studies, hypothesis testing and a multivariate-penalized logistic regression model (*firthlogit*), based on the Akaike information criterion (AIC).

**RESULTS:**

A total of 3573 students completed the survey, 61.5% were female, 55.3% were young (aged 18–26 years), and 44.6% were adults (aged 27–59 years). The prevalence of ENDS use during the pandemic was 7.3%. Age was negatively associated with ENDS use, as young people had a higher likelihood of using these devices. The likelihood of ENDS use was negative among females (OR=0.38; 95% CI: 0.2–0.5). In contrast, it was positive in students with a history of psychoactive substance abuse and/or dependence (OR=3.59; 95% CI: 1.0–12.0), students who had tried conventional cigarettes (OR=5.12; 95% CI: 3.0–8.5), participants who smoked tobacco during the pandemic (OR=3.15; 95% CI: 2.3–4.2), those who studied virtually (OR=1.52; 95% CI: 1.0–2.3), participants who lived with other ENDS users (OR=3.86; 95% CI: 2.8–5.2) and students with negative perception of impacts on their mental health (OR=1.48; 95% CI: 1.1–1.9).

**CONCLUSIONS:**

Being male, aged <26 years, having a history of substance use, having tried conventional cigarettes, pandemic tobacco use, and having lived with other ENDS users, were the main factors associated with pandemic ENDS use. Studying the consumption habits of students in response to lifestyle changes, is fundamental for the formulation of strategies to reduce the development of addictive behaviors, especially in young students during the pandemic.

## INTRODUCTION

The COVID-19 pandemic^1^ has resulted in an emergency, which has influenced people’s lifestyles^2^ by including threats to their individual, social, and financial well-being^3^. During the pandemic, attention has been placed on mental health, including behavioral disorders^4^ with an emphasis on addictions and their role in substance use and abuse^5^, including those consumed through electronic nicotine delivery systems (ENDS). The World Health Organization (WHO) estimates that there are currently 367 million users of ENDS in the world, with electronic cigarettes being the most popular prototype^6^. The ENDS have changed the habit of consuming nicotine, tobacco, and other psychoactive substances (PS), especially among adolescents and young adults^7^.

Viewed as a construct, the WHO establishes that lifestyle^2^ is based on behavioral patterns determined by interactions between individual and social perspectives, and by socioeconomic and environmental living conditions. Living conditions represent the daily environment of individuals, where they live, act and work, thus able to exert impacts on health. In Colombia, the National Survey on Psychoactive Substance Use^8^ reported a prevalence of ENDS consumption in the last 30 days of 3.2% in people aged 18–65 years for 2019, finding in young people (aged 18–24 years) a prevalence of 1.7%. This poses adolescence and youth as vulnerable groups with probable and greater risks of presenting tobacco and psychoactive substance consumption, as well as questioning their coping capacity^9^, and represents the first step in determining factors associated with addictive behaviors in this population.

The motivations of young people that lead them to consume tobacco products may be linked to curiosity and pleasure^9^. However, the implementation of measures to contain the outbreak during the pandemic, such as confinement, led the population to face stressful situations that affected their mental health^5^. Evidence finds that disasters (traumatic, natural, or environmental) that impact a large part of a population are always accompanied by health consequences, including addictions, causing many people to consume substances (alcohol, tobacco/ ENDS, psychoactive substances) as alternatives to coping with emotional burdens resulting from massive contingencies^10^.

Although there are multiple neurobiological hypotheses and theories underlying the mechanisms by which a person becomes immersed in addictions, the self-medication hypothesis could explain this behavioral process during the pandemic^11^. It stipulates that addiction is a tangible manifestation of the inability to endure negative affective states. From this perspective, consumption represents attempts to ‘self-medicate’ high levels of stress. This hypothesis, which describes part of the multi-causal nature of substance use and abuse, added to research that has reported associations between exposure to mass contingencies and higher rates of tobacco use and nicotine dependence^12^. All interventions aimed at prevention of consumption and at modifying risk factors during the pandemic are justified, as health impacts may occur immediately after and then persist for long periods of time^10^.

To date, legislation in Colombia that seeks to regulate the sale and consumption of ENDS, especially among minors, is advancing. Those in favor of the anti-tobacco law, including ENDS, are asking scientific societies for recent data on prevalence and associated factors during the pandemic, as they are concerned that the impacts on mental health may lead to addictive behaviors or exacerbate them^13^. Therefore, the objective of this study was to determine associations between the ENDS use and the lifestyle of students from Higher Education Institutions (HEI) in Bogotá, Colombia, after the onset of the COVID-19 pandemic, characterizing sociodemographic conditions associated with consumption, and identifying age groups that were more influenced by the growing phenomenon of ENDS consumption in this context.

## METHODS

### Design

We conducted an analytical, retrospective, cross-sectional study, based on a self-administered online survey, conducted between 17 March and 3 October 2021, of undergraduate students enrolled in public or private HEIs authorized by the Colombian Ministry of Education^14^. This research is part of a macro project (Prevalence and psychosocial factors associated with ENDS consumption in university populations of Bogotá) approved by the Corporate Research Ethics Committee of the Santa Fe Foundation^15^. This project is still in process.

### Sampling

With an estimation error of 5%, an estimated prevalence of 16%^16^ and adjusting for 20% losses, the sample size was 3985 students. A multistage, probability sampling proportional to the sample size, was used. The first stage used the list of institutions located in Bogotá as a source of information. The second stage was the random selection of undergraduate programs for each institution. All, students enrolled in the selected degree programs were candidates to answer the survey.

### Recruitment

Each HEI selected during the first stage was contacted to request their consent. In order to avoid reductions in the sample size and guarantee levels of precision and reliability for statistical analysis, a replacement was made in the event that any HEI selected refused to participate, taking an HEI immediately before or after the one originally selected in the sampling frame.

### Collection

Students received graphic pieces with information about the project and the link to the survey through their institutional email, at least three times. Once the participants gave their digital consent, they proceeded to fill out the instrument. Thirty items corresponding to the demographic characterization of the students, history of tobacco/ENDS use, and items related to consumption and lifestyle during the pandemic, were analyzed. Data were collected and stored in RedCap, with Stata (version 17) being the statistical software of choice.

According to the classification of the Colombian Ministry of Health regarding life cycles, people are considered young if they are aged between 14 and 26 years, and adults if they are aged between 27 and 59 years. In Colombia, any person, regardless of their age, can enroll in academic programs offered in higher education institutions. Undergraduate students who completed the survey and were aged ≥18 years were included in the study.

The study was approved by the Ethics Committee of the Fundación Santa Fe de Bogotá (FSFB), selected and financed by the Colombian Ministry of Science, Technology and Innovation, and executed by the Mental Health Department and the Population Health Axis of the FSFB22.

### Statistical analysis

To determine associations between ENDS consumption and students’ lifestyle during the pandemic, frequency studies were performed for descriptive statistical analysis. Since the dependent variable was dichotomous, bivariate analyses were performed using chi-squared and Fisher tests, determining associations between the independent variables and the dependent variable. Given the prevalence of students who claimed to have consumed ENDS during the pandemic, a penalized likelihood estimation method (Firth’s method) was used to reduce small sample bias in the maximum likelihood estimation. Bivariate analyses (*firthlogit*) were performed between the dependent variable and each independent variable. Finally, a multivariate-penalized logistic regression model (*firthlogit*)^17,18^ was constructed with the plausible variables that obtained a p<0.05 in the bivariate analysis. The final model chosen was based on a variable selection algorithm based on the Akaike information criterion (AIC)^19^. All the results of the analyses were provided as odds ratio (OR) or adjusted OR (AOR), presented with 95% confidence intervals (CI). A p<0.05 was considered statistically significant.

### Dependent variable

The primary outcome of interest gave insight into the question: ‘during the pandemic, did you use e-cigarettes?’ (Yes/No).

### Independent variables

These included responds to the lifestyle^9^ of the students, from individual perspectives^17^ [(age, gender (female/male), health affiliation (contributory/subsidized/special), HEI, self-reported history of mental and/or emotional pathology, presence of symptoms compatible with depression or anxiety, history (tobacco/ENDS use)], social perspectives^17^ [(economic level, residence, coexistence with other ENDS users, academic environment (virtual/blended/distance/displaced)], and work conditions (telework/semi-presential, face-to-face, new job, stopped working), during the pandemic.

## RESULTS

**Figure 1:**
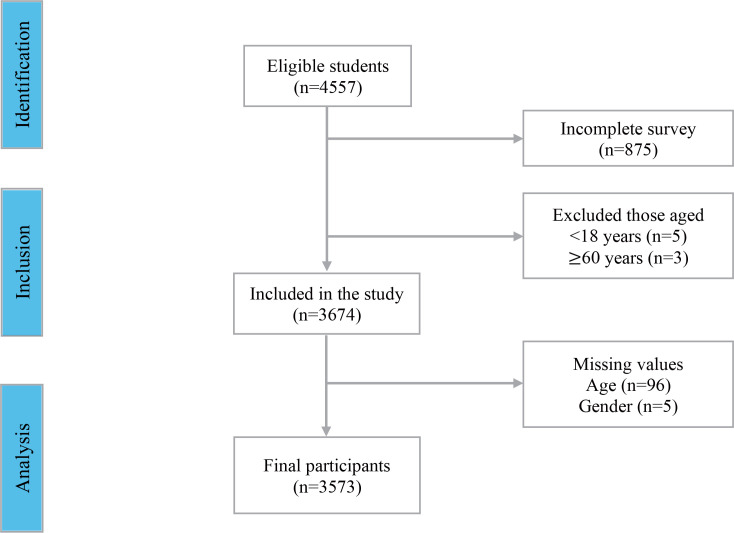
Students included in the data analysis

A total of 4557 students accepted to participate (80.7% completed the survey). Given the inclusion criteria, 8 students whose ages were outside the range were excluded, and 101 participants who erroneously completed the survey were also excluded (Figure 1). Nineteen authorized HEIs participated: 89.4% private and 10.6% public; 15.0% of the students were in their first semester, 12.3% in their sixth, seventh and eighth semesters, and 4.0% in their tenth semester or more. The age range was between 18 and 59 years, with a mean of 27 years and a 50th percentile of 25. According to the classification of the Colombian Ministry of Health regarding life cycles^20,21^, 55.3% of students were young (aged 18–26 years) and 44.6% were adults (aged 27–59 years). More than 50% of the sample were women (61.5%) (Table 1), and 63.8% of students reported having tried cigarettes at least once. Of those, 35% had smoked at least 100 cigarettes in their lifetime, characterizing them as current, non-experimental users. Females (55.2%) and youth (50.6%) had tried cigarettes more frequently; however, male adults (57.8%) represent a higher proportion of non-experimental smokers. The prevalence of ENDS use among students during the pandemic was 7.3% (Table 1). However, of the total sample, 29.9% reported having tried them at least once in their lifetime. Of these, 10.7% tried them between the ages of 12 and 15 years, 27% between the ages of 19 and 21 years, and 35.3% between the ages of 16 and 18 years. Among the participants who have tried ENDS, 44.5% have used them in the last year. Of them, 16% use them daily and 15.6% some days a week. Among the frequent users of ENDS in the last year, 9.7% had added another type of substance to the devices, with cannabis (97.8%) being the psychoactive substance most used by the students. Students were asked about their risk perception when comparing ENDS to cigarettes; 35.3% of the sample considered ENDS equally harmful, 10.5% thought these devices were more dangerous than cigarettes, 24% did not know the degree of risk, and 30.1% expressed that ENDS are less harmful.

**Table 1:** Characteristics of the students, distribution by age group and ENDS consumption during the pandemic, Bogotá, Colombia, 2021

*Characteristics*	*Age group 1*	*Age group 2*	*ENDS consumers during the pandemic*	
	*18–26 years*	*27–59 years*	*Yes*	*No*
	*(n=1977; 55.3%) n (%)*	*(n=1596; 44.6%) n (%)*	*(n=261; 7.3%) n (%)*	*(n=3312; 92.7%) n (%)*
**Gender**				
Male	712 (36)	661 (41.4)	171 (65.5)	1202 (36.2)
Female	1265 (63.9)	935 (58.5)	90 (34.4)	2110 (63.7)
**Economic level**				
0–2	659 (33.3)	703 (44)	27 (10.7)	1334 (40.2)
3–4	1038 (52.5)	843 (52.8)	148 (56.7)	1733 (52.3)
5–6	251 (12.6)	41 (2.5)	80 (30.65)	212 (6.4)
**Type of health affiliation**				
Contributory	1143 (57.8)	1260 (78.9)	156 (59.7)	2247 (67.8)
Subsidized	398 (20.1)	181 (11.3)	30 (11.4)	549 (16.5)
Special^a^	116 (5.8)	112 (7.0)	21 (8.0)	207 (6.2)
Unaffiliated	47 (2.3)	28 (1.7)	5 (1.9)	70 (2.1)
**Residence in Bogotá**				
Always	1149 (58.1)	646 (40.4)	159 (60.9)	1636 (49.4)
Never	262 (13.2)	501 (31.3)	19 (7.2)	744 (22.4)
**Self-reported history of mental and/or emotional pathology**				
Depressive disorder	161 (8.1)	85 (5.3)	31 (11.8)	215 (6.4)
Anxiety disorder	190 (9.6)	65 (4.0)	41 (15.7)	214 (6.4)
PS abuse and/or dependence^b^	9 (0.4)	4 (0.2)	6 (2.3)	7 (0.2)
None	1679 (84.9)	1457 (91.2)	204 (78.1)	2932 (88.5)

ENDS: electronic nicotine delivery system.

a Special: armed forces, eco-patrol, public universities, teachers, among others.

b PS: psychoactive substances

Among the associations between ENDS use and independent variables that responded to students’ lifestyle from individual and social perspectives during the pandemic (Table 2), it stands out that students who lived with other ENDS users during confinement were more likely to use ENDS (OR=5.6; 95% CI: 4.3–7.5). In general, symptoms consistent with depression and/or anxiety showed significant associations with ENDS use.

**Table 2:** Bivariate associations between student lifestyle and pandemic ENDS consumption, Bogotá, Colombia 2021

*Characteristics*	*ENDS consumers during the pandemic*			
	*Yes*	*No*	*Associations of ENDS consumers*	
	*(n=261; 7.3%) n (%)*	*(n=3312; 92.7%) n (%)*	*OR (95% CI)*	*p*
**Academic environment**				
Virtual	229 (87.4)	2719 (82.1)	1.5 (1.0–2.2)	0.02
Blended	25 (9.5)	156 (4.7)	2.1 (1.4–3.3)	<0.0001
**Work environment**				
Telework	51 (19.5)	837 (25.2)	0.7 (0.5–0.9)	0.03
Semi-presential	9 (3.4)	353 (10.6)	0.3 (0.1–0.6)	<0.0001
In-person	24 (9.2)	606 (18.6)	0.4 (0.3–0.7)	<0.0001
New job	35 (14.4)	204 (6.1)	2.3 (1.6–3.4)	<0.0001
Stopped working	23 (8.8)	494 (14.9)	0.5 (0.3–0.8)	<0.0001
**COVID-19 positive diagnosis**				
Individual	70 (26.8)	665 (20.0)	1.4 (1.1–1.9)	<0.0001
Family/friends	205 (78.5)	2310 (69.7)	1.5 (1.1–2.1)	0.003
**Economic difficulties**				
Yes	134 (51.3)	1965 (59.3)	0.7 (0.5–0.9)	0.012
**Co-existence with other ENDS users during the pandemic**				
Yes	95 (36.4)	303 (9.1)	5.6 (4.3–7.5)	<0.0001
**Perception on physical or mental impact during the pandemic**				
Mental health negative	158 (60.5)	1554 (46.9)	1.7 (1.3–2.3)	<0.0001
Mental health positive	36 (13.7)	325 (9.8)	1.4 (1.0–2.1)	0.04
Physical health negative	107 (41.0)	1201 (36.2)	1.2 (0.9–1.5)	0.12
Physical health positive	42 (16.0)	411 (12.4)	1.3 (0.9–1.9)	0.08
None	38 (15.5)	875 (26.4)	0.4 (0.3–0.6)	<0.0001
**Symptoms compatible with depression/anxiety**				
Sadness/hopelessness	189 (68.9)	1696 (51.2)	2.1 (1.6–2.7)	<0.0001
Anxiety	129 (49.3)	930 (28.0)	2.5 (1.9–3.2)	<0.0001
Loss of interest	163 (62.4)	1218 (36.7)	2.5 (2.2–3.6)	<0.0001
Sleep disturbances	169 (64.7)	1618 (48.8)	1.9 (1.4–2.4)	<0.0001

ENDS: electronic nicotine delivery system.

Notably, young students were the leading users of ENDS (78.1%) and cigarettes (57.4%) compared to adults during the pandemic. Among the active ENDS users, some students were found to be dual users with cigarettes (45.9%) and psychoactive substance (16.8%) during this period.

During the pandemic, almost twice as many students smoked tobacco (13.9%) compared to ENDS users (7.3%); while 38.8% reported having decreased their tobacco use and 27.6% did not change their tobacco consumption habits. In contrast, a smaller percentage of students who reported using ENDS decreased their tobacco use (27.2%). However, the tobacco users were those who, in greater proportion, increased (26.8%) their consumption compared to ENDS users who also reported having increased (20.3%) their use of these devices. Despite the higher number of students who smoked cigarettes in comparison to ENDS users, an important finding of this study is that the users of these devices were those who in a greater proportion reported having started (16.4%) their consumption habit during the pandemic, compared to students who reported having started (6.8%) to smoke tobacco.

Data showed that 37.9% of the students who used ENDS during the pandemic never thought about quitting. In contrast, 28.3% quitted temporarily and 14.9% quitted permanently. The reasons given by the participants for temporary or definite cessation of ENDS included that these were more difficult to obtain (30.9%) during the pandemic and that the use of ENDS made them more prone to infection by COVID-19 (32.9%). Emotional symptoms compatible with possible depression or anxiety were found in 81% of the sample. More than 52.5% of the respondents reported sadness, hopelessness and irritability, and 50% expressed sleep disturbances.

Finally, the multivariate regression model (Table 3) found that age was negatively associated with ENDS use during the pandemic (AOR=0.9; 95% CI: 0.8–0.9), with younger students being more likely to use ENDS. Females had 62% less chance of using ENDS than males (AOR=0.38; 95% CI: 0.2–0.5). Positive associations were found among students who reported a history of psychoactive substances abuse/dependence (AOR=3.59; 95% CI: 1–12). The odds of using ENDS were 3.8 times greater for students who lived with other ENDS users (AOR=3.86; 95% CI: 2.8–5.2). Students who reported having tried tobacco-related products in their lifetime (AOR=5.12; 95% CI: 3–8.5), and those who smoked tobacco during the pandemic (AOR=3.15; 95% CI: 2.3–4.2) were more likely to use ENDS during the pandemic. This increased likelihood was also found in participants who studied virtually (AOR=1.52; 95% CI: 1–2.3) and students with a perception of negative impacts on their mental health (AOR=1.48; 95% CI: 1.1–1.9).

**Table 3:** Factors associated with ENDS use during the pandemic among university students, Bogotá, Colombia 2021

*Factors*	*Multivariate model*		
	*OR (95% CI)*	*AOR (95% CI)*	*p*
Age (years)	0.9 (0.8–0.9)	0.90 (0.8–0.9)	<0.0001
Gender (Female)	0.3 (0.2–0.3)	0.38 (0.2–0.5)	<0.0001
History of abuse and/or dependence on psychoactive substances	3.6 (3.1–4.0)	3.59 (1.0–12)	0.043
Have you ever tried cigarettes?	8.2 (5.1–13)	5.12 (3.0–8.5)	<0.0001
Studied in virtuality during the pandemic	1.3 (1.0–1.7)	1.52 (1.0–2.3)	0.047
Perception of negative impact on mental health	1.7 (1.3–2.2)	1.48 (1.1–1.9)	0.008
Coexistence with other ENDS users during the pandemic	5.6 (4.3–7.5)	3.86 (2.8–5.2)	<0.0001
Cigarette consumption in pandemic	6.5 (5.0–8.5)	3.15 (2.3–4.2)	<0.0001

ENDS: electronic nicotine delivery system. AOR: adjusted odds ratio; adjusted for the variables of interest.

## DISCUSSION

This study sought to establish the prevalence of ENDS consumption, as well as the changes in this habit since the COVID-19 pandemic. The main findings revealed that, during the pandemic, the prevalence of ENDS use among students of HEIs in Bogotá was higher than results reported in national surveys for the year 2019^8^ in Colombia. Being male, aged 18–26 years, having a history of psychoactive substance abuse/dependence, having tried cigarettes, having a perception of negative impact on mental health, having studied virtually, and having lived with other ENDS users during the confinement, represented associated factors for ENDS consumption during the pandemic.

### Transformations in ENDS consumption habits during the pandemic

More students decreased and quitted than those who initiated or increased their ENDS use. These findings are consistent with Sokolovsky et al.^22^, who found that the frequency of smoking and vaping among university students decreased in the pandemic context. Shivani et al.^23^ also detailed that, since the health emergency began, almost twice as many respondents reported having abandoned ENDS use, compared to those who reported having increased their ENDS use. These findings show that the pandemic may have triggered multiple transformations in students’ tobacco and ENDS use behavior in response to changes in their lifestyles from individual and societal perspectives that are distinctive to the pandemic.

### Individual and social perspectives

Men had higher tobacco and ENDS use rates, which is similar to results from the national survey on psychoactive substances use^8^ and the World Bank reports^24^. Gender differences may be linked to physiological, cultural and behavioral factors. While men could be motivated by pleasure and taste, women are more likely to be motivated by stress relief and weight control^25^. In contrast, Hopkins et al.^26^ found that women were more likely to vape in the context of confinement, which was attributed to parenting and stressors such as uncertainty during the pandemic. In any case, both men and women were vulnerable to the lifestyle changes that occurred during the crisis1. Focusing attention on the motivations that led them to consume during confinement is relevant to the implementation of gender-specific consumption mitigation strategies.

The highest percentage of students who have ever used ENDS tried them between the ages of 16 and 18 years, which is relevant because people who start vaping at an early age are more likely to transition to conventional cigarettes^6^ and to develop addictive behaviors^11^, perhaps becoming smokers at risk of suffering the well-known health burdens associated with smoking^5^. Non-Communicable Diseases (NCDs)^5^ currently represent the leading cause of preventable deaths in the world, burden health systems, reduce people’s productivity and are strongly associated with a population’s poverty rate^5^, with tobacco use being the leading preventable risk factor.

Inquiring about student mental health during the pandemic, it was found that although a high percentage of vapers denied a history of mental/emotional pathology prior to the onset of the pandemic, many students reported having at least one adverse mental or behavioral health condition, including symptoms associated with anxiety disorders or depression, consistent with students experiencing symptoms such as mood disorders, sadness, sleep disturbances, appetite changes, loss of interest, and anxiety, with significant associations identified between these symptoms and ENDS use during the pandemic.

It is worth considering at this point that the likely negative impact on mental health has created new and stressful barriers to community living, especially for people already suffering from mental illness, substance use disorders and tobacco dependence^27^. It is estimated that, because of the confinement, there is a higher rate of anxiety and depression disorders, associated with lower mental wellbeing, compared to the usual and present before the contingency^27^. This is consistent with what was reported by the Center for Disease Control and Prevention (CDC), which stated that mental disorders increased considerably between April and June 2020, compared to the same period in 2019; results that become important because people who reported using psychoactive substances did so to cope with stress and emotions related to COVID-19^28^.

Regarding the perception of risk associated with the initiation or cessation of ENDS consumption, some respondents acknowledged not knowing the degree of risk of ENDS versus tobacco products. Despite the fact that studies establish that ENDS are mainly used to quit smoking^29^, the evidence shows that smokers have knowledge about smoking and, to a lesser extent about ENDS, but no perception of the risks. This is relevant as the concept of risk is eminently social and, therefore, the individual assumes the risks of the population where he/she lives^30^. Consequently, the perception of risk becomes a focal point of attention, since it has the capacity to assume protective measures for the individual, hence the importance of having information on the subject in times of crisis, which allows for effective interventions in favor of the health of the younger population. This is in line with students who reported having temporarily or permanently stopped using ENDS during the pandemic, attributing this to their personal perception of virus transmission, stating that vaping would make them more susceptible to COVID-19 infection.

Significant associations between students who used ENDS and lived with other ENDS users during confinement were observed, which is important because people’s smoking behavior and ENDS could be explained from social perspectives^31^. Evidence suggests that family dynamics, where at least one member is a smoker, are a predictor of the initiation of tobacco and ENDS use, especially among young people. However, the influence exerted by the family group is minor, compared to the influence exerted by peers, finding a greater individual susceptibility to the maintenance of addictive behaviors^32^.

Finally, significant associations were identified between virtuality and students’ ENDS consumption during the pandemic. This is congruent with the Sokolovsky et al.^22^ findings suggesting that students underwent changes in their consumption habits in response to the closure of the university campus, in order to continue in the virtual world. Social isolation as a measure to contain the spread of the virus^6^ and the consequent deployment of information technologies, exposed many people to stressful situations related to negative mental health outcomes^6^ and may represent associated factors that could lead to transformations in their lifestyles^33^, especially in behaviors related to the habit of ENDS during confinement.

Prevention programs targeting young people should include evidence-based information that allows them to understand the potential risks of ENDS on their health, as well as to become aware of advertising campaigns. The study of consumption patterns in response to lifestyle changes during the pandemic represents a valuable tool for policy makers to plan interventions that ensure adequate information dissemination to people at risk of addictive behaviors through health warnings aimed at mitigating consumption in times of pandemic.

### Limitations

The evidence presented is of utmost importance to understand the current prevalence of ENDS in university students and its association with the pandemic. However, being a cross-sectional study, causality between variables cannot be determined. In addition, the generalization of these results is limited to the population of students from participating HEIs located in Bogotá, which also recognizes the overrepresentation of private institutions compared to public ones in the study. Due to the differences between enrollment ages in Colombia in comparison to other countries, and the very wide age group included in our study, it could be possible that these results may not be comparable to other student populations.

Moreover, given the low prevalence of pandemic ENDS use, it was not feasible to capture in the final multivariate regression model all plausible variables that could have explained the response variable. All data collected are self-reported and this could affect the reliability of the information, especially when it comes to reporting behaviors for which there is social stigma (consumption: tobacco/ENDS/psychoactive substance) leading to social desirability biases. However, it should be noted that the survey was designed and implemented during the social isolation phase, with items constructed specifically to address factors associated with ENDS consumption habits, minimizing recall bias, which is the first step in understanding the effects of the pandemic on vaping behaviors.

## CONCLUSIONS

This study examined the associations between ENDS consumption habit and the emerging lifestyle of Bogotá HEIs students in the aftermath of the COVID-19 pandemic. With a prevalence of ENDS consumption of 7.3%, it was identified that being male, aged 18–26 years, having a history of psychoactive substance abuse/dependence, having tried cigarettes, using tobacco during the pandemic, having a perception of negative impact on mental health, having studied virtually, and having lived with other vapers during the confinement, were factors associated with the consumption of ENDS in times of health crisis. However, it was also found that a percentage of students found the pandemic an opportunity to quit tobacco and ENDS consumption temporarily or permanently.

## Data Availability

The data supporting this research are available from the authors on reasonable request.
